# Co‐occurrence models fail to infer underlying patterns of avoidance and aggregation when closure is violated

**DOI:** 10.1002/ece3.9104

**Published:** 2022-07-11

**Authors:** Robert C. Lonsinger

**Affiliations:** ^1^ U.S. Geological Survey, Oklahoma Cooperative Fish and Wildlife Research Unit Oklahoma State University Stillwater Oklahoma USA

**Keywords:** bias, closure, co‐occurrence, interspecific interaction, occupancy, simulation

## Abstract

Advances in multi‐species monitoring have prompted an increase in the use of multi‐species occupancy analyses to assess patterns of co‐occurrence among species, even when data were collected at scales likely violating the assumption that sites were closed to changes in the occupancy state for the target species. Violating the closure assumption may lead to erroneous conclusions related to patterns of co‐occurrence among species. Occurrence for two hypothetical species was simulated under patterns of avoidance, aggregation, or independence, when the closure assumption was either met or not. Simulated populations were sampled at two levels (*N =* 250 or 100 sites) and two scales of temporal resolution for surveys. Sample data were analyzed with conditional two‐species occupancy models, and performance was assessed based on the proportion of simulations recovering the true pattern of co‐occurrence. Estimates of occupancy were unbiased when closure was met, but biased when closure violations occurred; bias increased when sample size was small and encounter histories were collapsed to a large‐scale temporal resolution. When closure was met and patterns of avoidance and aggregation were simulated, conditional two‐species models tended to correctly find support for non‐independence, and estimated species interaction factors (SIF) aligned with predicted values. By contrast, when closure was violated, models tended to incorrectly infer a pattern of independence and power to detect simulated patterns of avoidance or aggregation that decreased with smaller sample size. Results suggest that when the closure assumption is violated, co‐occurrence models often fail to detect underlying patterns of avoidance or aggregation, and incorrectly identify a pattern of independence among species, which could have negative consequences for our understanding of species interactions and conservation efforts. Thus, when closure is violated, inferred patterns of independence from multi‐species occupancy should be interpreted cautiously, and evidence of avoidance or aggregation is likely a conservative estimate of true pattern or interaction.

## Graphical abstract

Advances in multi‐species monitoring have prompted an increase in the use of multi‐species occupancy analyses to assess patterns of co‐occurrence among species, even when data were collected at scales likely violating the assumption that sites were closed to changes in occupancy state for the target species. Occurrence for two hypothetical species were simulated under patterns of avoidance, aggregation, or independence, when the closure assumption was either met or not. When closure was violated, models tended to incorrectly infer a pattern of independence and power to detect simulated patterns of avoidance or aggregation decreased with smaller sample size, which could have negative consequences for our understanding of species interactions and conservation efforts.

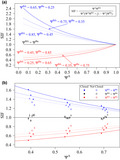

## 1 INTRODUCTION

Understanding the environmental factors and interspecific interactions driving patterns of species occurrence underpins the field of community ecology (Morin, 2011) and can provide critical insights to improve the conservation of biological communities, and mitigate negative consequences of global change (e.g., urbanization, invasive species, and climate; Singer et al., 2013). Occupancy modeling uses species‐specific detection/non‐detection data collected over repeat surveys of sites to estimate a species' probability of occurrence (Ψ; hereafter, occupancy), while accounting for imperfect detection (MacKenzie et al., 2002). Inclusion of site‐specific covariates can allow identification of environmental factors associated with patterns of occurrence, whereas survey‐specific covariates can be used to address heterogeneity in detection. Extensions to occupancy modeling have facilitated inferences about the relative influences of environmental factors and interspecific interactions on species' patterns of occurrence (MacKenzie et al., 2021; Richmond et al., 2010; Rota et al., 2016). Conditional two‐species models assume that two species interact through an asymmetrical relationship and the occurrence of a dominant species may influence the occurrence of a subordinate species (but not vice versa; Richmond et al., 2010). By contrast, unconditional multi‐species occupancy modeling does not make any a priori assumptions about the dominance of sympatric species, and can be used to investigate interactions among two or more species (MacKenzie et al., 2021; Rota et al., 2016).

Single‐season, single‐species occupancy models assume that sites are closed to changes in an occupancy state for the target species over a sampling season (i.e., the period over which repeat surveys are conducted across all sites; Mackenzie, 2005a, MacKenzie et al., 2018). This assumption is required in order to account for imperfect detection. When the closure assumption is violated, occupancy estimates tend to be biased high, which could have negative consequences for species conservation (Devarajan et al., 2020; Rota et al., 2009). The ability to satisfy the closure assumption is influenced (in part) by the correspondence between the spatial ecology of the target species and the study design, including the spatial and temporal scales used to define a site and season, respectively. For example, sites that are small relative to the movement capacity of the target species, or surveys spanning a season that is long relative to the target species’ seasonal movement patterns or survival, may lead to violations of the closure assumption. When the closure assumption is violated and movements in and out of sites are random, estimates of occupancy may be interpreted as an unbiased estimate of ‘use’ (i.e., the probability that the species used a site during the sampling season; Gould et al., 2019; Mackenzie, 2005a, 2005b). If the proportion of sites occupied is a primary interest (e.g., as a state parameter or surrogate for abundance; MacKenzie & Nichols, 2004), estimates of ‘use’ may be uninformative or lead to erroneous conclusions about the state of the population (Mackenzie & Royle, 2005). Still, estimates of ‘use’ can provide valuable information on wildlife‐habitat associations and relative habitat quality (Gould et al., 2019).

Extensions to occupancy modeling to investigate patterns of co‐occurrence for >1 species—conditional two‐species (Richmond et al., 2010) and multi‐species (Rota et al., 2016) occupancy modeling—maintain the same assumptions as single‐species occupancy modeling (MacKenzie et al., 2002), including the assumption that sites are closed to changes in an occupancy state over a sampling season (i.e., the closure assumption). As with single‐species occupancy modeling, the assumption of closure is necessary to estimate the probability of detection (*p*) for each species, but may also influence inferred patterns of co‐occurrence. For example, consider a site surveyed four times during a single season for two species (Species A and Species B), where the encounter histories (*h*) for Species A and Species B are *h* ^A^ = 1000 and *h* ^B^ = 0001, respectively. If the closure assumption is met in this example, it is clear that both species co‐occurred at this site. By contrast, if closure is not met, it is unclear if the two species co‐occurred, or if each species used the site when the other was absent.

A common goal of modeling co‐occurrence is to evaluate if the occurrence of one species influences the occurrence of another species. Closure may be more difficult to meet in studies investigating patterns of co‐occurrence for >1 species, particularly when species differ in their spatial ecology (e.g., home range size, movement tendencies, and density), as the assumption of closure applies to each species considered in the analysis. While failing to meet the closure assumption in single‐species analyses shifts the interpretation of Ψ to ‘use’, it is unclear how this influences the ability to infer true patterns of co‐occurrence. Nevertheless, studies explicitly acknowledging violations of the closure assumption have gone on to use multi‐species detection histories to infer patterns of co‐occurrence (e.g., Li et al., 2019; Staudenmaier et al., 2021).

Here, I simulated occurrence for two hypothetical species (Species A and Species B) under co‐occurrence patterns of avoidance, aggregation, or independence, when the closure assumption either was or was not met during sampling. I then evaluated if conditional two‐species occupancy models provided support for the true co‐occurrence pattern, and if the inferences changed when sampling intensity was reduced or when the encounter history was discretized to a larger temporal resolution (i.e., when multiple sequential surveys were collapsed into longer survey occasions). I hypothesized that when the closure assumption was met, (i) two‐species occupancy models would tend to provide support for the true pattern of co‐occurrence, (ii) reductions in sampling intensity would lead to less consistency in support for the true pattern of co‐occurrence, and (iii) a large‐scale temporal resolution of the encounter history would not impact model performance. By contrast, I hypothesized that when the closure assumption was violated, (i) two‐species occupancy models would tend to find support for independence even when the true pattern of co‐occurrence was avoidance or aggregation, and (ii) this tendency to find support for independence would be increased by reductions in sampling intensity and using large‐scale temporal resolution encounter histories.

## 2 MATERIALS AND METHODS

### 2.1 Simulation conditions

I simulated patterns of occurrence for two hypothetical species (Species A and Species B) across a simple continuous landscape comprised of 900 equal‐sized grid cells without environmental variation. I assumed that Species A was dominant and Species B was subordinate (sensu Richmond et al., 2010). For simplicity, I assumed that occupancy of a site by either species was by a single individual and, therefore, there was no abundance‐induced heterogeneity in *p*. I randomly distributed Species A across the landscape such that true occupancy of Species A (Ψ^A^) was 0.4, 0.55, or 0.7 (Table 1). I then distributed Species B in three scenarios in relation to Species A: (i) the occurrence of Species B was negatively associated with the occurrence of Species A (i.e., avoidance); (ii) the occurrence of Species B was positively associated with the occurrence of Species A (i.e., aggregation); or (iii) the occurrence of Species B was not influenced by the occurrence of Species A (i.e., independence). For simulations of avoidance, Species B was randomly distributed across the landscape such that true occupancy of Species B, given that Species A was present (Ψ^BA^), and true occupancy of Species B, given that Species A was absent (Ψ^Ba^), was 0.25 and 0.65, 0.35 and 0.75, or 0.45 and 0.85, respectively (Table 1). For simulations of aggregation, Species B was randomly distributed across the landscape such that Ψ^BA^ and Ψ^Ba^ were 0.65 and 0.25, 0.75 and 0.35, or 0.85 and 0.45, respectively (Table 1). For simulations of independence, Species B was randomly distributed across the landscape such that Ψ^B^ (i.e., Ψ^BA^ = Ψ^Ba^) was 0.45, 0.55, or 0.65 (i.e., the average values of Ψ^BA^ and Ψ^Ba^ combinations were used for simulations of avoidance or aggregation; Table 1).

**TABLE 1:** True values of occupancy for Species A (Ψ^A^) and Species B (Ψ^BA^ = occupancy when influenced by and in the presence of Species A; Ψ^Ba^ = occupancy when influenced by and in the absence of Species A; Ψ^B^ = occupancy of when independent of Species A) used to simulate species occurrence under two patterns of influence (avoidance where Ψ^BA^ < Ψ^Ba^ and aggregation where Ψ^BA^ > Ψ^Ba^) and a pattern of independence (where Ψ^B^ = Ψ^BA^ = Ψ^Ba^)

	Avoidance		Aggregation		Independence
Ψ^A^	Ψ^BA^	Ψ^Ba^	Ψ^BA^	Ψ^Ba^	Ψ^B^
0.4	0.25	0.65	0.65	0.25	0.45
0.4	0.35	0.75	0.75	0.35	0.55
0.4	0.45	0.85	0.85	0.45	0.65
0.55	0.25	0.65	0.65	0.25	0.45
0.55	0.35	0.75	0.75	0.35	0.55
0.55	0.45	0.85	0.85	0.45	0.65
0.7	0.25	0.65	0.65	0.25	0.45
0.7	0.35	0.75	0.75	0.35	0.55
0.7	0.45	0.85	0.85	0.45	0.65

I evaluated the performance of conditional two‐species occupancy models when the closure assumption was either (i) met or (ii) not met. To evaluate model performance when closure was met, I generated 500 simulated patterns of co‐occurrence for each combination of occupancy parameters, maintaining the initial distributions of Species A and Species B during sampling. To evaluate model performance when closure was not met (i.e., species could move among sites between surveys), I again generated 500 simulated patterns of co‐occurrence for each combination of occupancy parameters, which served as the initial distribution. For simplicity, I assumed (i) both species had the capacity to move among sites between surveys, (ii) the probability of movement (*M*) from an occupied site to another site between surveys was the same for both species (*M* ^A^ = *M* ^B^ = *M*), and (iii) *M* = 0.02, a level selected to represent a relatively low probability of closure violations. Between surveys, I assumed movements by the dominant species (Species A) were independent of the subordinate species (Species B). For Species A, I randomized the order in which individuals were permitted to move between each survey and, when an individual moved from a site, I probabilistically selected the settlement site as a function of the site's availability (i.e., not currently occupied by the same species) and distance from the starting site, such that movements were more likely to nearby available sites. Although it was not possible for an individual to move to a site that was already occupied by the same species, a site that was occupied during the previous survey and vacated by an earlier departure could be selected as a settlement site. For simulations in which Ψ^BA^ = Ψ^Ba^, the same process was repeated to simulate movements for Species B. For simulations in which Ψ^BA^ ≠ Ψ^Ba^, I probabilistically selected the settlement site for Species B as a function of the site's availability, distance from the starting site, and Ψ^BA^ (when Species A was present at a site) or Ψ^Ba^ (when Species A was not present at a site).

For all simulations, I set *p* for Species A (*p* ^A^) and Species B (*p* ^B^) to 0.05 (i.e., *p* ^A^ = *p* ^B^ = 0.05) and assumed the probability of detection for each species was (i) independent of the presence or detection of the other species, and (ii) constant over time. I generated species‐specific encounter histories for *N* = 250 randomly sampled sites over *J* = 21 replicated surveys (hereafter, fine‐scale temporal resolution). The temporal resolution at which encounter data is considered may influence the ability to infer patterns of co‐occurrence that operate at temporal scales that are small relative to the survey length. To investigate how temporal resolution of surveys influenced the reliability of conditional two‐species modeling, I also collapsed surveys to *J* = 3 survey occasions, where each occasion contained 7 sequential surveys of the initial 21 surveys (hereafter, large‐scale temporal resolution). Camera traps inherently detect multiple species and have become a leading source of data for multi‐species occupancy modeling. Consequently, values for *p* and *J* were intended to emulate camera‐trap data collected over relatively short surveys (e.g., days) that could be discretized into longer survey occasions (e.g., weeks). The selected *p* was comparable to daily camera‐based detection probabilities reported for carnivores and ungulates (Shannon et al., 2014), and resulted in a cumulative probability of detection of 1–(1–0.05)^7^ = 0.30 across 7 surveys (i.e., a survey occasion) and 1 – (1–0.05)^21^ = 0.66 across all surveys. Finally, field logistics often restrict practitioners to sampling a relatively small proportion of the landscape. Consequently, I repeated all the simulation procedures and evaluated performance of conditional two‐species models when sample size was reduced from *N* = 250 to *N* = 100 randomly sampled sites. All simulations were performed in program R (R Core Team, 2021).

### 2.2 Modeling and assessing patterns of co‐occurrence

For each set of conditions, I analyzed the sample data using two competing single‐season, conditional two‐species models (Richmond et al., 2010): (i) a model in which the occurrence of Species A influenced the occurrence of Species B (i.e., Ψ^A^, Ψ^BA^ ≠ Ψ^Ba^; where Ψ^BA^ and Ψ^Ba^ were estimated separately); and, (ii) a model for independence between Species A and Species B (i.e., Ψ^A^, Ψ^BA^ = Ψ^Ba^; where Ψ^BA^ and Ψ^Ba^ were not estimated separately and were presented together as Ψ^B^). I held the detection submodels for each species constant (i.e., the null model), regardless of occupancy or detection state of the other species. I performed all occupancy analyses with an information‐theoretic approach in program R with the ‘wiqid’ package (Meredith, 2020). For each model set, I evaluated relative support for competing models with Akaike's Information Criterion with sample size correction (AIC_c_; Burnham & Anderson, 2002). I excluded models that failed to converge from subsequent summaries and comparisons. When the most‐supported model suggested that Species A influenced Species B (i.e., support for a model of influence), I calculated the SIF as

$$\mathrm{SIF}=\frac{\Psi^{A}\Psi^{\mathrm{BA}}}{\Psi^{A}\left(\Psi^{A}\Psi^{\mathrm{BA}}+\left[1-\Psi^{A}\right]\Psi^{\mathrm{Ba}}\right)}$$

following Richmond et al. (2010). A SIF <1 suggested avoidance (i.e., Species B occurred less frequently with Species A than expected compared to a null hypothesis of independence), whereas a SIF >1 suggested aggregation (i.e., Species B occurred more frequently with Species A than expected). A SIF = 1 suggested that the two species occurred independently of one another.

For each set of conditions, I summarized the reliability of the occupancy estimates from the most‐supported models across simulations. Percent bias of occupancy estimates relative to the true parameter values were calculated for each simulation with the ‘SimDesign’ package in R (Chalmers & Adkins, 2020). I summarized percent bias as the mean bias for each parameter and set of conditions. To characterize overall performance under each set of conditions, I summarized the proportion of simulations that found support for the true pattern of co‐occurrence. Additionally, I considered the model of independence as competitive when (i) the simulated pattern was independence, (ii) the model for a pattern of influence had the lowest AICc, and (iii) the model of independence (which had one fewer parameter than the model of influence) was within 2 ΔAICc units of the top model (Burnham & Anderson, 2002). Thus, for simulations of independence, I reported both the proportion of simulations supporting the true pattern of co‐occurrence and an adjusted proportion when considering competitive models finding support for independence. For each set of conditions, I calculated the expected (or true) SIF and then compared the mean SIF (across simulations when a model of influence was most supported) to the expected SIF.

## 3 RESULTS

The simulated landscape contained 900 cells oriented in a 30 × 30 grid. Reducing the sample size from *N* = 250 to *N* = 100 represented a reduction in sampling intensity from ~28% to ~11% of the simulated landscape. For each combination of simulated conditions, <0.4% of the 500 simulations failed to achieve numerical convergence.

**FIGURE 1:**
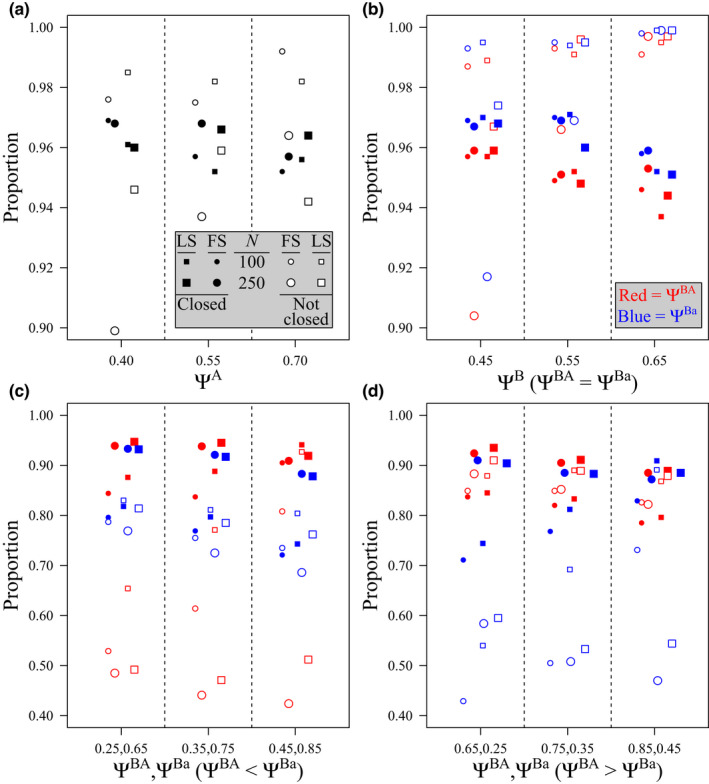
Proportion of simulations for which the estimates of (a) occupancy for Species A (Ψ^A^), (b) occupancy of Species B given Species A was present (Ψ^BA^) and occupancy of Species B, given that Species A was absent (Ψ^Ba^) when the simulated pattern was independence (i.e., Ψ^BA^ = Ψ^Ba^), (c) Ψ^BA^ and Ψ^Ba^ when the simulated pattern was avoidance (i.e., Ψ^BA^ < Ψ^Ba^), and (d) Ψ^BA^ and Ψ^Ba^ when the simulated pattern was aggregation (i.e., Ψ^BA^ > Ψ^Ba^), was not different from the true simulated level of occupancy (based on 95% confidence intervals) when sites were closed or not closed (i.e., open) to changes in the occupancy state during sampling with sampling at *N* = 100 or 250 random sites and repeated surveys at a fine‐scale (FS) or large‐scale (LS) temporal resolution. Results presented are based on the most‐supported conditional two‐species occupancy model

Based on the most‐supported model for each simulated dataset, estimates of Ψ^A^ were generally unbiased when the population was closed (mean bias <3.1%), and positively biased when the population was not closed (mean bias 7.3%–13.3%), with bias being higher when the sample size was small (*N =* 100), and encounter histories were collapsed to a large‐scale temporal resolution (Table 2). Across simulated conditions, a high proportion of Ψ^A^ estimates were not different from the true (simulated) value (i.e., the 95% confidence intervals (CIs) about the estimate included the true value; Figure 1a). Similarly, estimates of Ψ^BA^ and Ψ^Ba^ were generally unbiased when the population was closed (mean bias <5%), and biased when the population was not closed, with bias being higher when the sample size was small (Table 3). Under simulated patterns of independence, a high proportion of Ψ^B^ estimates were not different from the true value (Figure 1b). Precision of estimates tended to decrease when the sample size was small and when the closure assumption was violated. For example, when considering estimates of Ψ^A^ under simulated patterns of independence, reducing the number of sites sampled (while maintaining closure) or violating the closure assumption (while maintaining *N* = 250 sites sampled) resulted in a ~37% or ~17% increase in the width of 95% CIs about the estimate, respectively, while reducing the number of sites sampled and violating the closure assumption resulted in a ~49% increase in the width of 95% CIs. Consequently, a greater proportion of simulations had estimates with 95% CIs containing the true value when sample size was small, closure was violated, or both (Figure 1a,b). Under simulated patterns of avoidance and aggregation, the proportion of models with estimates for Ψ^BA^ and Ψ^Ba^ for which the 95% CI contained the true value was generally higher when the closure assumption was met and sample size was large (Figure 1c,d). By contrast, when closure was violated and the true value of Ψ^BA^ or Ψ^Ba^ was low (<0.5), relatively few models tended to produce estimates with 95% CIs containing the true value (Figure 1c,d).

**TABLE 2:** Mean percent bias (*B*) in estimates of occupancy for Species A across 500 simulations with three true levels of occupancy of Species A (Ψ^A^) under three patterns of co‐occurrence with Species B (i.e., independence, avoidance, and aggregation) when the population was either closed (Closed) or not closed (Open) to changes in the occupancy state with sampling at *N* = 100 or 250 random sites and repeated surveys at a fine‐scale (FS) or large‐scale (LS) temporal resolution (TR)

*N*	TR	Ψ^A^	Independence		Avoidance		Aggregation	
			(Ψ^BA^ = Ψ^Ba^)		(Ψ^BA^ < Ψ^Ba^)		(Ψ^BA^ > Ψ^Ba^)	
			Closed *B* ^A^	Open *B* ^A^	Closed *B* ^A^	Open *B* ^A^	Closed *B* ^A^	Open *B* ^A^
100	FS	0.40	2.17	8.95	1.32	8.72	1.45	9.29
		0.55	1.20	10.73	1.39	10.18	1.14	10.80
		0.70	1.26	10.35	0.07	10.04	0.88	10.53
100	LS	0.40	3.07	11.79	1.61	11.28	2.25	12.08
		0.55	1.64	13.18	1.75	12.59	1.31	13.30
		0.70	1.50	12.27	0.38	11.81	1.28	12.17
250	FS	0.40	0.68	7.41	0.78	7.33	0.72	7.64
		0.55	0.51	9.33	0.34	9.11	0.77	9.36
		0.70	0.49	10.24	0.26	9.52	0.43	9.88
250	LS	0.40	0.82	9.51	0.98	9.09	0.91	9.72
		0.55	0.84	11.74	0.58	11.07	0.89	11.60
		0.70	0.60	12.12	0.28	11.36	0.59	11.75

*Notes*: Ψ^BA^ = True occupancy of Species B in the presence of Species A; Ψ^Ba^ = True occupancy of Species B in the absence of Species A; Fine‐scale temporal resolution included 21 surveys; Large‐scale temporal resolution collapsed fine‐scale surveys into three surveys each containing seven sequential fine‐scale surveys.

**TABLE 3:** Mean percent bias (*B*) in estimates of occupancy for Species B across 500 simulations with three combinations of true occupancy of Species B in the presence (Ψ^BA^) or absence (Ψ^Ba^) of Species A under three patterns of co‐occurrence (i.e., independence, avoidance, and aggregation) when the population was either closed (Closed) or not closed (Open) to changes in occupancy state with sampling at *N* = 100 or 250 random sites and repeated surveys at a fine‐scale (FS) or large‐scale (LS) temporal resolution (TR)

*N*	TR	Independence (Ψ^BA^ = Ψ^Ba^)					Avoidance (Ψ^BA^ < Ψ^Ba^)					Aggregation (Ψ^BA^ > Ψ^Ba^)				
			Closed		Open			Closed		Open			Closed		Open	
		Ψ^B^	*B* ^BA^	*B* ^Ba^	*B* ^BA^	*B* ^Ba^	Ψ^BA^, Ψ^Ba^	*B* ^BA^	*B* ^Ba^	*B* ^BA^	*B* ^Ba^	Ψ^BA^, Ψ^Ba^	*B* ^BA^	*B* ^Ba^	*B* ^BA^	*B* ^Ba^
100	FS	0.45	1.67	1.06	9.42	8.80	0.25, 0.65	3.45	0.31	19.52	−2.03	0.65, 0.25	−1.14	4.42	−0.89	19.27
		0.55	1.46	2.22	11.18	10.24	0.35, 0.75	3.98	−1.27	21.47	−3.99	0.75, 0.35	−1.53	3.01	−1.68	21.23
		0.65	2.01	1.23	10.79	9.90	0.45, 0.85	4.52	−4.80	24.08	−6.83	0.85, 0.45	−3.85	4.87	−2.86	22.75
100	LS	0.45	2.24	1.67	11.95	11.35	0.25, 0.65	3.96	1.50	21.90	0.16	0.65, 0.25	−0.84	4.85	1.33	21.40
		0.55	2.00	2.81	13.62	12.28	0.35, 0.75	4.47	−1.14	24.06	−1.94	0.75, 0.35	−1.49	3.32	0.83	22.39
		0.65	2.82	1.87	12.67	11.62	0.45, 0.85	4.99	−4.57	26.36	−4.92	0.85, 0.45	−3.99	4.87	−1.27	23.81
250	FS	0.45	0.26	0.51	8.35	8.53	0.25, 0.65	0.51	1.60	15.56	2.76	0.65, 0.25	0.79	−0.03	0.13	13.38
		0.55	0.36	0.91	9.30	8.75	0.35, 0.75	0.74	1.78	18.54	−0.30	0.75, 0.35	−0.25	0.32	−0.32	16.42
		0.65	0.68	0.26	10.67	8.63	0.45, 0.85	0.86	−0.33	21.13	−3.17	0.85, 0.45	−0.60	1.18	−1.66	20.78
250	LS	0.45	0.40	0.71	10.56	10.69	0.25, 0.65	0.68	2.42	17.17	6.07	0.65, 0.25	1.12	0.02	2.48	13.46
		0.55	0.71	1.34	11.81	11.02	0.35, 0.75	0.95	2.55	20.29	2.16	0.75, 0.35	−0.01	−0.05	2.04	16.68
		0.65	0.95	0.53	13.03	10.60	0.45, 0.85	0.95	−0.13	23.24	−1.59	0.85, 0.45	−0.52	0.47	0.40	20.83

*Notes*: Ψ^B^ = True occupancy of Species B when the occurrence of Species A does not influence the occurrence of Species B (i.e., Ψ^BA^ = Ψ^Ba^); Fine‐scale temporal resolution included 21 surveys; Large‐scale temporal resolution collapsed fine‐scale surveys into three surveys each containing seven sequential fine‐scale surveys.

**FIGURE 2:**
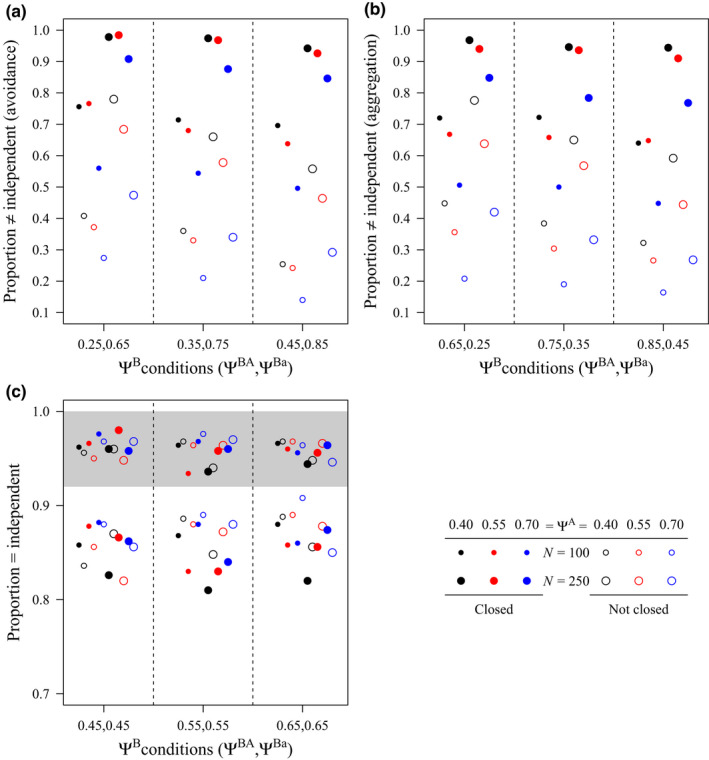
Proportion of simulations for which conditional two‐species occupancy models supported the true simulated pattern of co‐occurrence between Species A and Species B when sites were closed or not closed (i.e., open) to changes in occupancy state during sampling, and sampling occurred at more (*N* = 250) or fewer (*N* = 100) sites; simulations were performed under various levels of true occupancy for Species A (Ψ^A^) and patterns of co‐occurrence for Species B (Ψ^B^) including (a) a pattern of influence (i.e., avoidance) in which the true occupancy of Species B, given that Species A was present (Ψ^BA^) was lower than the true occupancy of Species B, given that Species A was absent (Ψ^Ba^), (b) a pattern of influence (i.e., aggregation) in which Ψ^BA^ > Ψ^Ba^, and (c) a pattern of independence (i.e., Ψ^BA^ = Ψ^Ba^). Results within the gray bar represent the adjusted proportion when considering competitive models within 2 ΔAIC_c_ finding support for independence. Results are presented based on encounter histories at a fine‐scale resolution

Of primary interest was whether conditional two‐species models tended to recover the simulated pattern of co‐occurrence. Similar patterns in model performance emerged for both patterns of co‐occurrence in which Species A influenced Species B. Conditional two‐species models tended to correctly find support for an influence of Species A on Species B when the closure assumption was met and sample size was large, with the proportion of models identifying the correct pattern being slightly higher for patterns of avoidance (Figure 2a) than aggregation (Figure 2b). By contrast, models tended to incorrectly find support for independence between Species A and Species B when the closure assumption was violated and sample size was small (Figure 2a,b). Generally, models were less likely to correctly identify a simulated pattern of influence as the true value of Ψ^A^ increased (Figure 2a,b). When the simulated pattern of co‐occurrence between Species A and Species B was independence, conditional two‐species models tended to correctly infer independence. Moderate increases in performance (identification of a true pattern of independence) tended to occur with smaller sample size, violations of the closure assumption, and higher values of Ψ^A^ and Ψ^B^ (Figure 2c). Across simulated conditions, the proportion of model sets correctly identifying independence increased when considering those where the model of influence was most supported, but the model of independence was competitive (Figure 2c; Burnham & Anderson, 2002). Although Figure 2 presents results based on encounter histories at a fine‐scale resolution, results based on large‐scale encounter histories were similar (results not plotted).

**FIGURE 3:**
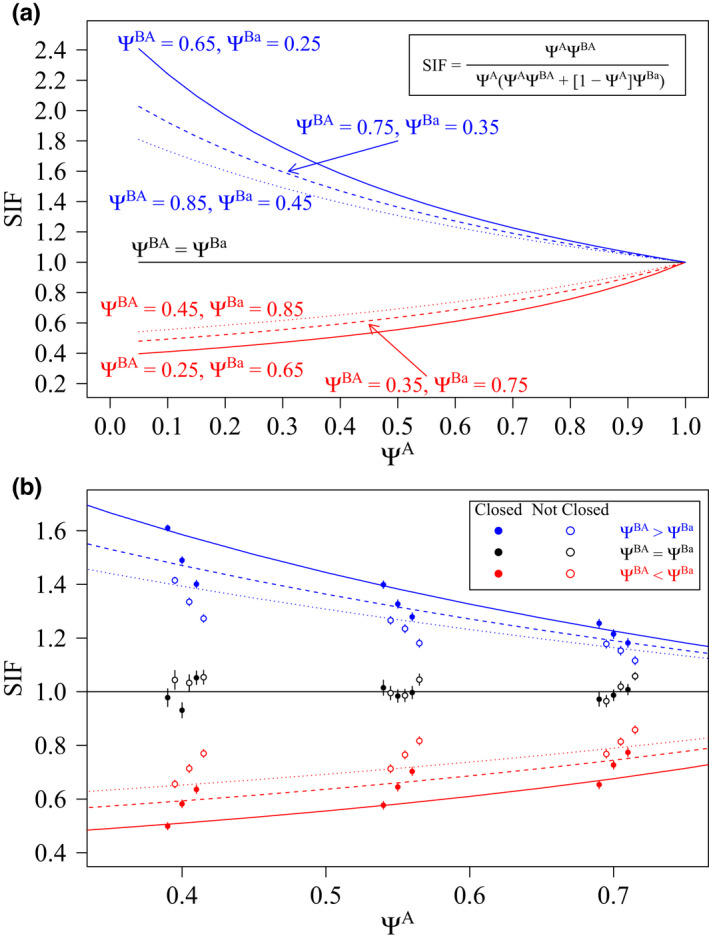
(a) Expected species interaction factor (SIF) for simulated combinations of occupancy for Species B, given that Species A was present (Ψ^BA^) or absent (Ψ^Ba^) from a site under patterns of avoidance (red), aggregation (blue), or independence (black) as a function of the occupancy for Species A (Ψ^A^); and, (b) mean estimated SIF (±1 standard error) for simulations where the most‐supported model provided support for a lack of independence (i.e., avoidance or aggregation) when sites were closed (solid circles) or not closed (open circles) to changes in occupancy state and sampling occurred at *N* = 250 sites. For each simulated level of Ψ^A^ when sites were closed (or not), results for combinations of Ψ^BA^ and Ψ^Ba^ are presented from left to right by increasing the value of Ψ^BA^. Results are presented based on encounter histories at a fine‐scale resolution

When the simulated pattern was avoidance or aggregation, and the conditional two‐species model found support for an influence of Species A on Species B, the estimated SIF values aligned with the expected values based on the simulated patterns (Figure 3). The magnitude of the estimated SIF tended to suggest a weaker interaction between Species A and Species B when the closure assumption was violated (Figure 3). When the simulated pattern was independence and the conditional two‐species model found support for an influence, the mean estimated SIF values aligned with independence (Figure 3). The SIF results presented here (and in Figure 3) were based on sampling *N* = 250 sites with fine‐scale encounter histories. When sample size was reduced to *N* = 100, patterns were similar but there was greater variability in the mean estimated SIF, and the magnitude of the estimated SIF suggested a stronger interaction between Species A and Species B (results not plotted). Collapsing fine‐scale encounter histories to a large‐scale temporal resolution had negligible impacts on SIF results.

## 4 DISCUSSION

Understanding co‐occurrence patterns among sympatric species is of broad ecological interest and has implications for the conservation of imperiled species and ecosystems. Multi‐species occupancy models offer effective approaches for inferring patterns of co‐occurrence (MacKenzie et al., 2018) and have been used to investigate ecological patterns and theories (Lombardi et al., 2020; Robinson et al., 2014), interactions among native and invasive species (Dugger et al., 2016; Hegel et al., 2019; Osorio et al., 2020), and community response to global change (Parsons et al., 2019; Sovie et al., 2020). The within‐season closure assumption inherent to occupancy modeling (MacKenzie et al., 2002) extends to multi‐species models as well (Devarajan et al., 2020; Richmond et al., 2010; Rota et al., 2016), but the impact of violating the closure assumption on inferences about patterns of co‐occurrence has not been evaluated. My results suggest that when the closure assumption is violated, occupancy‐based models of co‐occurrence often fail to detect underlying patterns of avoidance or aggregation and incorrectly identify a pattern of independence among species, which could have negative consequences for the conservation of biodiversity.

Camera trapping has been one of the most used sampling techniques for occupancy‐based investigations of multiple species (Burton et al., 2015; O'Connell et al., 2011). Rapid advancements in camera technology (e.g., reliability and capabilities) and the increasingly widespread use of cameras has facilitated a new era of multi‐species monitoring (Allan et al., 2018; Nazir et al., 2017; O'Connell et al., 2011). Additionally, large‐scale camera‐based monitoring programs that offer open‐source multi‐species data streams have recently been established (e.g., SnapshotUSA; Cove et al., 2021). Consequently, while the opportunity to apply multi‐species occupancy models will likely increase, data collection procedures may not be at the correct spatial and temporal scales necessary to meet the closure assumption for many species. The simulations presented here emulated camera trapping data and the simulated level for detection was comparable to observed levels of daily detection for several carnivores (Shannon et al., 2014). Like the conditions under which the simulated data were collected and discretized, camera trap data is commonly characterized as many short surveys (e.g., daily encounter histories; Parsons et al., 2019) or fewer long surveys (e.g., weekly encounter histories; O'Connell et al., 2006). Still, results from the simulations here based on the large‐scale temporal resolution in which surveys were collapsed into three survey occasions, would be more comparable to data from alternative sampling approaches (e.g., visual encounter or call‐back surveys) where fewer surveys are typically completed and a cumulative ‘occasion‐level’ detection probability of ~0.3 would not be unreasonable (e.g., Mackenzie & Royle, 2005).

Closure is influenced by the length of the season (i.e., the period over which surveys are performed), spatial scale of a site (i.e., the unit of analysis), and spatial ecology of the target species (MacKenzie et al., 2018). Even with careful consideration, it may be difficult to meet the closure assumption for some species (Mackenzie & Royle, 2005) due to logistical constraints (e.g., limited equipment or personnel) that require sampling to take longer than a period over which closure can be reasonably assumed (e.g., Johnson et al., 2020), or challenges identifying an appropriate spatial scale (e.g., Jornburom et al., 2020). Consequently, the closure assumption is often relaxed with estimates of occupancy from single‐species models interpreted as the probability of ‘use’ (Gould et al., 2019; Mackenzie, 2005a). For multi‐species occupancy analyses, the challenge of meeting the closure assumption is amplified, as the assumption applies to all target species, and species may operate at different spatial and temporal scales. When the closure assumption is violated, species‐specific estimates of occupancy tend to be biased high (Devarajan et al., 2020; Rota et al., 2009, but see Kendall et al., 2013 and Otto et al., 2013 for conditions where lack of closure may lead to negative bias in occupancy estimates), and this would likely lead to greater perceived overlap in the distributions of sympatric species than actually occurred, potentially limiting the ability of co‐occurrence models to detect patterns of non‐independence (Steen et al., 2014). Indeed, my results demonstrated that when closure was violated, conditional two‐species models produced estimates of occupancy that were biased high for both species, and models were less likely to detect true patterns of avoidance or aggregation. The ability to detect patterns of avoidance or aggregation requires variation in occurrence of both species. As demonstrated in Figure 3, the expected SIF tends toward (and converges at) 1 as Ψ^A^ increases to 1, regardless of the Ψ^BA^:Ψ^Ba^ ratio. This suggests that when estimates of Ψ^A^ are high—whether naturally or due to an upward bias resulting from a lack of closure—patterns of avoidance and aggregation will be more difficult to detect. My results support these predictions, with the power to detect patterns of avoidance and aggregation decreasing for higher values of Ψ^A^ and when closure was violated.

MacKenzie and Royle (2005) and Shannon et al. (2014) provided recommendations for the optimal number of sites for single‐species occupancy modeling, but how sample size influences the ability to infer patterns of co‐occurrence has not been investigated. I initially simulated data collection at 250 sites, a sampling intensity that approximated the optimal number of sites (Mackenzie & Royle, 2005) based on the simulated number of surveys, detection, and average occupancy, with a standard error of 0.06. In practice, sampling intensity is often limited (e.g., Robinson et al., 2014 [83], Nagy‐Reis et al., 2017 [45], Osorio et al., 2020 [50]). Consequently, I also considered data collection at only 100 sites to reflect a restricted sampling intensity that more closely aligned with many empirical studies. As expected, conditional two‐species models had greater power to detect patterns of avoidance and aggregation when sample size was larger.

Emmet et al. (2021) suggested that discretizing continuous‐time species detections could lead to sparse data and underestimates of detection, inflating estimates of occupancy. Similarly, I expected that collapsing fine‐scale temporal surveys into longer survey occasions would inflate occupancy estimates, and thus decrease the power to detect patterns of avoidance or aggregation. I observed higher bias in estimates when using a longer survey occasion versus the fine‐scale temporal resolution; this pattern occurred regardless of sample size or whether closure was met, though the magnitude of the effect was lower for the larger sample size and with closure. Still, in contrast to expectations, collapsing encounter histories from a fine‐scale to a large‐scale temporal resolution had no noticeable impact on the power to detect patterns of avoidance or aggregation or mean SIF estimates.

Collectively, these findings suggest that closure violations could lead to erroneous support for patterns of independence among species when the true pattern of co‐occurrence is avoidance or aggregation. Several studies have explicitly acknowledged violations of the closure assumption, used conditional two‐species occupancy models to assess patterns of co‐occurrence, and then concluded that the species occurred independently of one another (e.g., Li et al., 2019; Staudenmaier et al., 2021). In these scenarios, my results suggest that the inferred patterns of independence could be an artifact of a lack of closure and should be interpreted cautiously. By contrast, when closure violations likely occur and models find evidence of avoidance (e.g., Kafley et al., 2019) or aggregation, my results suggest the inferred pattern (and associated SIF) is likely a conservative estimate of the interaction (Steen et al., 2014). When closure violations may be obscuring patterns of co‐occurrence (i.e., at the site level), careful interpretation of detection parameters or temporal activity (e.g., from survey methods such as cameras that provide information on activity) may provide evidence of within‐site spatial or temporal patterns of interspecific interactions (e.g., Lewis et al., 2015; Lonsinger et al., 2017).

Minimizing closure violations may involve design‐based solutions or model‐based solutions, or both. Ideally, sampling designs for multi‐species occupancy modeling should consider the research question(s) and ecological traits of target species (e.g., patterns of phenology and movement), and employ appropriate spatial and temporal scales such that violations of the closure assumption are minimized (see Devarajan et al., 2020 for an excellent discussion of best practices for multi‐species occupancy modeling). Integrating knowledge on target species' ecologies to develop an a priori sampling design that minimizes closure violations will reduce the need to rely on complicated modeling procedures to address bias in estimates and inferences (Devarajan et al., 2020; Kendall et al., 2013; MacKenzie et al., 2018; Otto et al., 2013). Selecting sampling sites that approximate the home‐range size of the target species may reduce closure violations, as can conducting surveys as quickly as possible within and across sites (Mackenzie & Royle, 2005). However, differences in life‐history traits among species can make it difficult to identify an appropriate joint sampling design for all target species (Devarajan et al., 2020). System‐specific simulations considering the disparate life‐history traits of target species and potential forms of closure violations (e.g., random movement, staggered entry and exit, or local extinction due to sampling disturbance) can help inform sampling design, reduce bias in parameter estimates, and improve inferences on species interactions (Devarajan et al., 2020; Kendall et al., 2013; Otto et al., 2013). When using open‐source data streams (or other similar data sources) for which the original sampling design was not intended for multi‐species occupancy modeling, the scale of data collection and appropriate scale for co‐occurrence modeling may be misaligned (Altwegg & Nichols, 2019). When closure violations are expected (or known to occur) for one or more species under an implemented sampling design, evidence of closure violations should be tested for and, if detected, accounted for through model‐based solutions to reduce bias and improve inferences on patterns of co‐occurrence.

Assessing closure can be difficult. Nonetheless, evaluating if the closure assumption is met for each target species can provide guidance on the suitability of the data, offer greater insights into the strength of inferences related to patterns of co‐occurrence, or both. Goodness‐of‐fit tests for occupancy models can detect evidence for violations of model assumptions (Broms et al., 2016; MacKenzie & Bailey, 2004; Warton et al., 2017), but (i) often fail to provide meaningful results when sample size is small (i.e., fail to detect poor fit when it occurs; MacKenzie & Bailey, 2004), and (ii) can suggest adequate model fit even when closure violations occur via random movements (Gould et al., 2019). If multiple surveys are conducted within each site visit (or sampling occasion) during a season, dynamic occupancy analyses (Kendall et al., 2013; Otto et al., 2013; Rota et al., 2009), which apply Pollock's robust design (Pollock, 1982) and estimate colonization and extinction (i.e., dynamic parameters) between site visits, can be extended for multiple species (Farris et al., 2017; Fidino et al., 2019; MacKenzie et al., 2021) and compared to static (single‐season) models to formally test for evidence of closure (e.g., via a likelihood ratio test). Although the application of dynamic models is more appropriate than goodness‐of‐fit tests for assessing closure, the approach requires a sampling design that is not commonly used for occupancy modeling (or anticipated to be used for many future studies) due to the extra effort required (Kendall et al., 2013). Alternatively, species‐specific occurrence data may be temporally thinned (or partitioned) and analyzed as smaller temporal intervals to check if the signs (effects) of beta coefficients changed (Devarajan et al., 2020). Time‐dependent single‐season models may not be adequate for reducing bias caused by closure violations (Otto et al., 2013).

The scenarios evaluated here were not exhaustive and simulations performed were intentionally simplified to minimize the factors influencing inferences about patterns of co‐occurrence. Nevertheless, these simulations highlight challenges to interpretation of results from co‐occurrence models when closure violations occur, and similar challenges are expected under other scenarios unless closure violations are minimized. The extent of closure violation was directly related to the probability of movement, which was fixed at a low rate such that the probability that the species never moved from a site was >0.65. Consequently, the results of these simulations reflect expectations when closure violations are relatively low, and more severe violations of the closure assumption would likely further decrease the power of co‐occurrence models to detect patterns of avoidance or aggregation. Simulations not presented here indicated that for the average values of occupancy used in this study, decreasing the probability of movement for both species from 0.02 to 0.005 increased the proportion of simulations for which the true pattern of avoidance (0.90) or aggregation (0.87) was detected, whereas increasing the probability of movement to 0.05 decreased the proportion of simulations for which the true pattern of avoidance (0.31) or aggregation (0.25) was detected. I did not consider conditions where closure was violated for a single species. Although beyond the scope of the current work, simulations evaluating the influence of environmental covariates, closure violations by only a single species, and additional sampling design considerations would help advance our understanding of factors impacting the reliability of inferred co‐occurrence patterns. Although I restricted the simulations to two species and analyzed the data with the widely used conditional two‐species occupancy modeling framework, the application of unconditional multi‐species occupancy models (Rota et al., 2016) has been increasing. I expect violations of the closure assumption would have similar impacts on results from unconditional multi‐species modeling (Rota et al., 2016), including underestimates of detection, overestimates of occupancy, and higher perceived overlap in the distribution of species than actually occurred, which would limit the power to detect patterns of non‐independence. Still, simulations evaluating the impacts of closure on inferences from unconditional modeling procedures and considering >2 species would advance our understanding of the reliability of inferences related to species co‐occurrence patterns.

## AUTHOR CONTRIBUTIONS

**Robert Lonsinger:** Conceptualization (lead); data curation (lead); formal analysis (lead); investigation (lead); methodology (lead); project administration (lead); resources (lead); writing – original draft (lead); writing – review and editing (lead).

## CONFLICT OF INTEREST

The author declares no conflict of interest or competing interests.

## Data Availability

All code used to generate simulations and perform analyses has been archived in a GitLab repository at https://doi.org/10.5066/P9H788YI (Lonsinger, 2022).
